# Charting Cervical Spinal Cord Morphometry Across the Lifespan

**DOI:** 10.1002/hbm.70658

**Published:** 2026-10-21

**Authors:** Kurt G. Schilling, Michael E. Kim, Matthew Amandola, Chenyu Gao, Karthik Ramadass, Praitayini Kanakaraj, Sam Bogdanov, Gaurav Rudravaram, Nancy R. Newlin, Derek Archer, Timothy J. Hohman, Angela L. Jefferson, Victoria L. Morgan, Alexandra Roche, Dario J. Englot, Murat Bilgel, Lori L. Beason Held, Luigi Ferrucci, Laurie E. Cutting, Laura A. Barquero, Micah A. D'archangel, Tin Q. Nguyen, Kathryn L. Humphreys, Yanbin Niu, Sophia Vinci‐Booher, Carissa J. Cascio, The HABS‐HD Study Team, Alzheimer's Disease Neuroimaging Initiative, The BIOCARD Study Team, Sid E. O'Bryant, Kristine Yaffe, Arthur Toga, Robert Rissman, Leigh Johnson, Meredith Braskie, Kevin King, James R. Hall, Melissa Petersen, Raymond Palmer, Robert Barber, Yonggang Shi, Fan Zhang, Rajesh Nandy, Roderick McColl, David Mason, Bradley Christian, Nicole Phillips, Stephanie Large, Joe Lee, Badri Vardarajan, Monica Rivera Mindt, Amrita Cheema, Lisa Barnes, Mark Mapstone, Annie Cohen, Amy Kind, Ozioma Okonkwo, Raul Vintimilla, Zhengyang Zhou, Michael Donohue, Rema Raman, Matthew Borzage, Michelle Mielke, Beau Ances, Ganesh Babulal, Jorge Llibre‐Guerra, Carl Hill, Rocky Vig, Zhiyuan Li, Daniel Moyer, Simon N. Vandekar, Panpan Zhang, Samuelle St‐Onge, Sandrine Bédard, Jan Valošek, Benjamin De Leener, Julien Cohen‐Adad, John C. Gore, Seth Smith, Bennett A. Landman

**Affiliations:** ^1^ Department of Radiology and Radiological Sciences Vanderbilt University Medical Center Nashville Tennessee USA; ^2^ Vanderbilt University Institute of Imaging Science Nashville Tennessee USA; ^3^ Department of Computer Science Vanderbilt University Nashville Tennessee USA; ^4^ Department of Electrical and Computer Engineering Vanderbilt University Nashville Tennessee USA; ^5^ Medical Scientist Training Program Vanderbilt University Nashville Tennessee USA; ^6^ Vanderbilt University Medical Center, Vanderbilt Memory and Alzheimer's Center Nashville Tennessee USA; ^7^ Vanderbilt University Medical Center Vanderbilt Genetics Institute Nashville Tennessee USA; ^8^ Vanderbilt University, Vanderbilt Brain Institute Nashville Tennessee USA; ^9^ Vanderbilt Alzheimer's Disease Research Center Nashville Tennessee USA; ^10^ Department of Medicine Vanderbilt University Medical Center Nashville Tennessee USA; ^11^ Department of Neurology Vanderbilt University Medical Center Nashville Tennessee USA; ^12^ Department of Psychology and Human Development Vanderbilt University Nashville Tennessee USA; ^13^ Department of Psychiatry and Behavioral Sciences Vanderbilt University Medical Center Nashville Tennessee USA; ^14^ Department of Biomedical Engineering Vanderbilt University Nashville Tennessee USA; ^15^ Department of Neurological Surgery Vanderbilt University Medical Center Nashville Tennessee USA; ^16^ Laboratory of Behavioral Neuroscience National Institute on Aging, National Institutes of Health Baltimore Maryland USA; ^17^ Department of Special Education Peabody College of Education and Human Development Nashville Tennessee USA; ^18^ Life Span Institute and Department of Psychology University of Kansas Lawrence Kansas USA; ^19^ Department of Computer Science Park University Parkville Missouri USA; ^20^ Department of Biostatistics Vanderbilt University Medical Center Nashville Tennessee USA; ^21^ NeuroPoly Lab Institute of Biomedical Engineering, Polytechnique Montréal Montréal Quebec Canada; ^22^ CHU Sainte‐Justine Research Centre Montréal Quebec Canada; ^23^ Spinal Cord Injury Center Balgrist University Hospital, University of Zürich Zürich Switzerland; ^24^ Departments of Neurosurgery and Neurology, Faculty of Medicine and Dentistry Palacký University Olomouc Olomouc Czechia; ^25^ Department of Biomedical Engineering University Hospital Olomouc Olomouc Czechia; ^26^ Centre de recherche du CHU Sainte‐Justine Montréal Canada; ^27^ Department of Computer Engineering and Software Engineering Polytechnique Montréal Montréal Canada; ^28^ Functional Neuroimaging Unit, CRIUGM Université de Montréal Montreal Quebec Canada; ^29^ Mila—Quebec AI Institute Montreal Quebec Canada

## Abstract

Spinal cord morphometry provides essential biomarkers of neurological health, but clinical interpretations are confounded by inter‐subject variability and a lack of normative references across the full human lifespan. We address this gap by generating the first comprehensive lifespan charts for cervical spinal cord morphometry. From 78,269 T1‐weighted brain MRI sessions, we identified a final cross‐sectional cohort of 24,251 healthy or cognitively normal controls from 33 datasets (ages 0–100 years) with cervical cord coverage. To overcome contrast variability, we employed a state‐of‐the‐art contrast‐agnostic deep learning segmentation method, extracting cross‐sectional area (CSA), anteroposterior (AP) and right–left (RL/transverse), and shape indices (AP/RL ratio, eccentricity, and solidity) from C1 to C7. Normative trajectories were modeled using Generalized Additive Models for Location, Scale, and Shape (GAMLSS). The resulting charts reveal distinct non‐linear lifespan changes: rapid growth through childhood and adolescence, peak maturation occurring in early‐to‐mid adulthood (e.g., mid‐30s for CSA), followed by gradual decreases. Significant regional variations along the cervical cord and consistent sex differences (males > females for size metrics) were quantified. Spinal cord trajectories showed strong temporal coupling with brain white matter and brainstem volumes, suggesting integrated CNS development and aging. These lifespan charts provide a robust normative framework, enabling age‐ and sex‐specific centile scoring of individual spinal cord morphometry. This resource offers a critical tool for differentiating typical variation from pathological changes, enhancing the clinical utility of spinal cord MRI in studies of development and neurodegeneration.

## Graphical abstract

From large multi‐site lifespan data, we segment C1–C7 and derive morphometric measures (area, diameters, shape). Normative centile charts summarize population trajectories across age and sex and provide a practical scoring framework to quantify where an individual lies relative to expected variation.

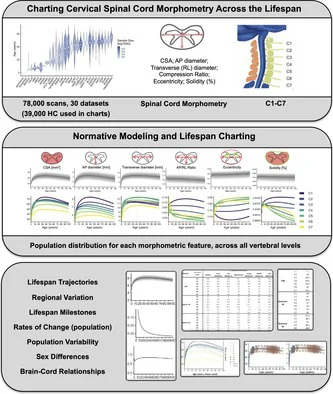

## 1 Introduction

Spinal cord morphometry provides quantitative measures reflecting macrostructural integrity that are directly relevant to neuroscience and clinical care (Bedard and Cohen‐Adad 2022; Bedard, Karthik, et al. 2025; Frostell et al. 2016; Horakova et al. 2022). Cross‐sectional area (CSA), anteroposterior (AP) diameter, and right–left (RL; transverse) diameter quantify cord size and are commonly used to characterize growth and atrophy. The AP/RL ratio (sometimes called “compression” ratio) describes flattening, eccentricity describes deviation from circularity, and solidity reflects contour indentation; these shape measures may capture focal deformation not reflected by cord size alone (Horakova et al. 2022; Muhammad et al. 2024; Bedard, Valošek, et al. 2025). These measures are altered across neurological conditions—including multiple sclerosis (Keegan et al. 2024; Mahajan et al. 2025), amyotrophic lateral sclerosis (Barry et al. 2022; Emmenegger et al. 2024; Lorenzi et al. 2020), spinal cord injury (Freund et al. 2013), and degenerative cervical myelopathy (Muhammad et al. 2024; Bedard, Valošek, et al. 2025)—and may support disease monitoring and prognosis. In multiple sclerosis, spinal cord atrophy is detectable from the earliest stages and correlates with motor disability (Bischof et al. 2022; Sastre‐Garriga et al. 2020; Ruggieri et al. 2021); in degenerative cervical myelopathy, automated morphometric profiling complements radiological assessment of stenosis and helps standardize detection of compressed cord (Muhammad et al. 2024; Bedard, Valošek, et al. 2025; Hohenhaus et al. 2024). To interpret these measurements and disentangle pathology from natural inter‐subject variation, a lifespan reference for normative values is needed.

Despite this clinical potential, broad adoption is limited by measurement variability that complicates subject‐specific interpretation and reduces statistical power in group studies. A century of reports—from post‐mortem measurements (Donaldson and Davis 1903; Elliott 1945) to modern MRI (Sherman et al. 1990; Joo et al. 2025; Valosek, Bédard, et al. 2024)—has yielded disparate “normative” values for spinal cord diameters and area (for a review, see [Frostell et al. 2016]), with several factors driving this inconsistency. First, biological sources such as age, sex, and individual anatomical differences produce between‐person dispersion (Bedard and Cohen‐Adad 2022; Papinutto et al. 2015; Papinutto, Asteggiano, et al. 2020; Rashid et al. 2006; Solstrand Dahlberg et al. 2020). Second, methodological sources introduce bias across studies, including scanner vendor, acquisition contrast/protocol, and analysis software assumptions about cord boundaries (Weeda et al. 2019; Lukas et al. 2021; Gros et al. 2019). Finally, variability arises from spatial localization, encompassing both the precision of identifying anatomical landmarks and the inherent neuroanatomical variance between vertebral levels and functional spinal cord segments (Bedard and Cohen‐Adad 2022). Together these effects hinder cross‐site aggregation, meta‐analysis, and reliable longitudinal tracking.

Normative modeling addresses these challenges by adapting the logic of pediatric growth charts (Kuczmarski et al. 2000; Grummer‐Strawn et al. 2010) to neuroimaging. Recent brain‐wide “charts” provide age‐ and sex‐stratified trajectories and variance across the lifespan (Bethlehem et al. 2022; Yang et al. 2024). Crucially, such models estimate the full population‐level distribution of a given biological measure—not just the mean—so individual measurements can be expressed as centile scores relative to age‐appropriate expectations, enabling standardized detection of atypical development or atrophy (Bethlehem et al. 2022).

The main barrier to creating comprehensive spinal cord charts has been the lack of large‐scale, dedicated imaging datasets spanning the full human lifespan. While valuable prior work laid the foundation by creating normative databases for specific populations, such as the PAM50 spinal cord template for adults (Joo et al. 2025; Valosek, Bédard, et al. 2024; De Leener et al. 2018), and by exploring normalization strategies to reduce inter‐subject variability in adult and pediatric cohorts (Bedard and Cohen‐Adad 2022; Muhammad et al. 2024; Bedard, Valošek, et al. 2025; Papinutto, Asteggiano, et al. 2020; Lee et al. 2015), these efforts were often limited by narrow age ranges, modest sample sizes, a focus on single vertebral levels, or simplified models unable to capture age‐varying variance. Here, we overcome these constraints by leveraging numerous large‐scale, population‐based brain neuroimaging datasets whose imaging protocols included cervical cord coverage. The feasibility and reliability of using such data for upper cervical cord morphometry are well‐established (Daams et al. 2014; Liu et al. 2016), presenting an exemplary opportunity to utilize existing data resources. However, aggregating data across these diverse cohorts also introduces significant methodological challenges, primarily MRI contrast variability (across sites or contrasts). To address this, we employ a recently developed, state‐of‐the‐art, contrast‐agnostic segmentation method (Bedard, Karthik, et al. 2025; Karthik et al. 2026), which provides consistent morphometric estimates across different scanners, sites, and protocols.

Our goal is to create vertebral‐level, sex‐stratified normative charts of cervical spinal cord morphometry across the lifespan. From an initial pool of 78,269 T1‐weighted MRI sessions, we assembled a cross‐sectional cohort of 24,251 healthy or cognitively normal controls from 33 neuroimaging datasets spanning ages 0–100 years. We (i) model the non‐linear trajectories of spinal cord growth, maturation, and atrophy; (ii) identify key lifespan milestones (e.g., age of peak CSA and diameters); (iii) quantify annualized (cross‐sectional) rates of change for clinical benchmarking across life stages; (iv) characterize population variability; (v) delineate sex‐specific trajectories; and (vi) relate cord morphometry to brain volumes across development and aging.

## 2 Materials and Methods

This study involved three main stages: (i) aggregation and curation of a large‐scale, multi‐site neuroimaging dataset; (ii) automated processing of all images to extract spinal cord and brain morphometric features; and (iii) normative modeling of these features to generate lifespan charts.

### 2.1 Study Cohorts

This work builds upon prior large‐scale data aggregation efforts that collated imaging from publicly available and locally acquired neuroimaging studies (Kim et al. 2026). The imported pool comprised 78,269 T1‐weighted MRI sessions from 48,130 dataset‐specific participants spanning ages 0–100 years.

We first retained sessions from participants designated by their source cohorts as healthy or cognitively normal controls, yielding 41,784 sessions from 26,697 participants. Visual quality assurance (see below) retained 37,888 sessions from 24,437 participants. After removing individuals with missing age/sex, we created a final cross‐sectional modeling dataset by randomly selecting a single scan from each participant with multiple time points, a strategy consistent with large‐scale charting initiatives (Bethlehem et al. 2022). This yielded a final cohort of 24,251 participants from 33 datasets (Figure 1A,B). Additional dataset information, sample sizes, and age ranges are provided in Table S1. T1w imaging parameters are given in Table S2.

**FIGURE 1:**
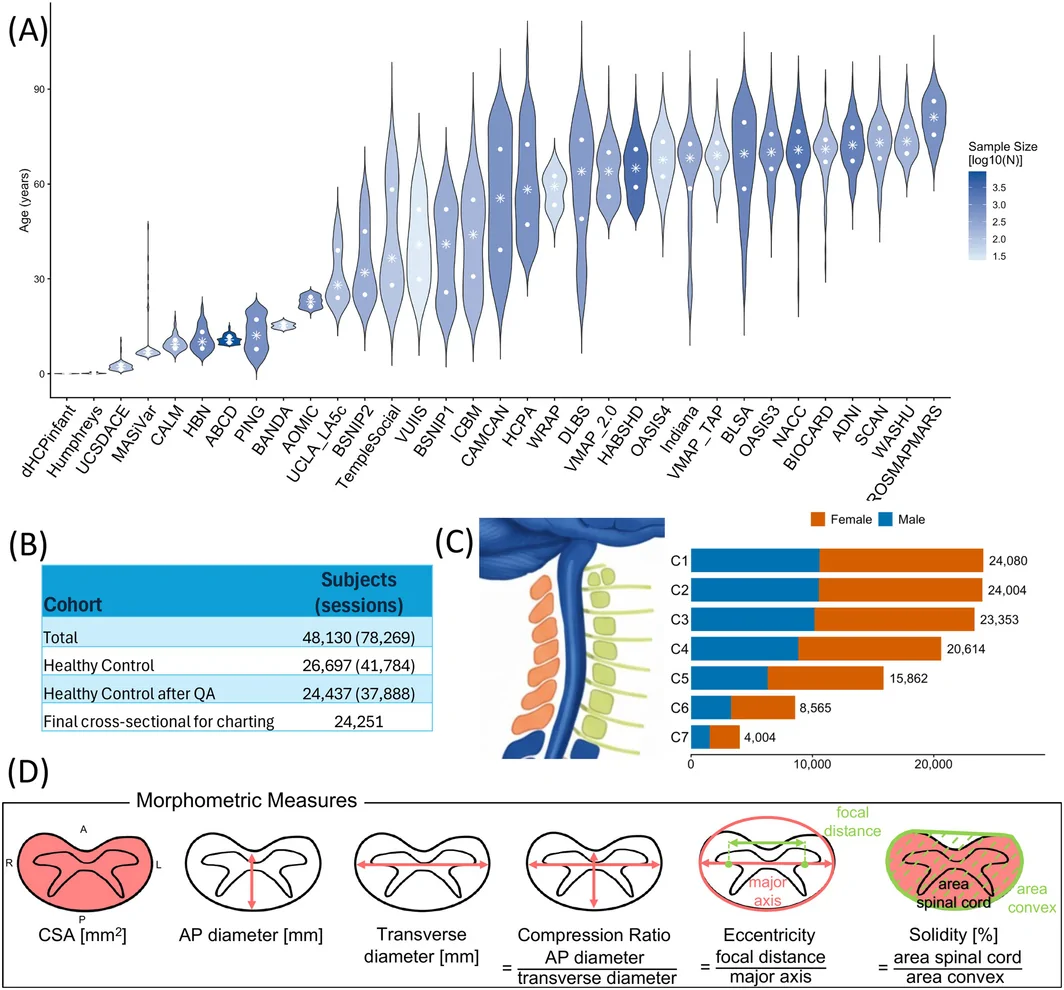
Overview of study cohorts and extracted spinal cord morphometry. Age distributions are shown across 33 contributing datasets for the final 24,251 participants used to create the spinal cord charts (A). Of 48,130 dataset‐specific participants (78,269 sessions), 26,697 participants (41,784 sessions) were designated healthy or cognitively normal controls, 24,437 participants (37,888 sessions) remained after scan‐level QA, and selection of one eligible scan per participant yielded 24,251 participants from 33 datasets for charting (B). The number of participants contributing to the CSA models varied by vertebral level (C). Six morphometric features were extracted at each available level: cross‐sectional area (CSA), anteroposterior (AP) diameter, right–left (RL) diameter, AP/RL ratio, eccentricity, and solidity (D; cord illustration recreated *from* Valosek, Bédard, et al. 2024).

All vertebral labeling and segmentation outputs underwent visual quality control (Figure S1); labeling failures were excluded and segmentation failures at the affected level only (i.e., failures were more common inferiorly), so a measurement contributed only when the corresponding level passed QA. Because cervical coverage varied across brain T1‐weighted acquisitions, the final sample sizes range from C1 = 24,080 to C7 = 4004 samples. (Figure 1C); their age‐by‐sex distributions are shown in Figure S2. We note that levels C6/C7 have sparse data throughout young adulthood.

### 2.2 Image Processing

#### 2.2.1 Spinal Cord Morphometry

All T1‐weighted images were processed with the Spinal Cord Toolbox (SCT, v7.1) (De Leener et al. 2017). To handle variability in image contrast across the aggregated datasets, we used a state‐of‐the‐art, contrast‐agnostic deep learning model to automatically segment the spinal cord from all T1‐weighted images (*sct_deepseg spinalcord*) (Bedard, Karthik, et al. 2025). This method offers a key advantage critical for this large‐scale study: it provides robust and consistent segmentation performance regardless of the input image contrast (i.e., differences in acquisition parameters across sites). Vertebral levels were extracted using a tool for automatic instance segmentation of all vertebrae (*sct_deepseg totalspineseg*), that is also robust to various MRI contrasts, acquisition orientations, and resolutions. Following SCT convention, C1 and C2 were retained as separate projected vertebral regions; because no intervertebral disc separates the atlas and axis, their boundary was estimated from upper‐cervical vertebral landmarks (Valosek, Bédard, et al. 2024; De Leener et al. 2017).

Following segmentation, three measures of cord size (CSA and AP‐ and RL‐diameters) and three measures of cross‐sectional shape (AP/RL ratio, eccentricity, and solidity) were extracted at each vertebral level in native space using *sct_process_segmentation* (Figure 1D):

- Cross‐sectional area (CSA) (mm^2^): The total area of the spinal cord in the axial plane (on a centerline‐orthogonal axial slice), calculated from the binary segmentation by summing the voxel‐wise probabilities. CSA is a primary indicator of spinal cord atrophy.
- Anterior–posterior (AP) diameter (mm): The maximum diameter of the spinal cord along the anterior–posterior axis. Calculated as the AP axis of a fitted ellipse.
- Right–Left (RL)/transverse diameter (mm): The maximum diameter of the spinal cord along the right–left axis, also referred to as the transverse diameter. Calculated as the RL axis of a fitted ellipse.
- AP/RL Ratio (a.u.): The ratio between the AP and RL diameters, which provides a measure of cord flattening (lower value indicates greater flattening). This had previously been referred to as “compression” ratio in literature and within SCT (Valosek, Bédard, et al. 2024), but is called AP/RL ratio here as its name may inappropriately suggest spinal cord compression.
- Eccentricity (a.u.): A measure of the spinal cord's deviation from a perfect circle, calculated from an ellipse with the same second‐order moments as the cord segmentation (range 0–1; values nearer 0 indicate a more circular cross‐section).
- Solidity: The ratio of the CSA to the area of its convex hull, which quantifies the convexity of the cord shape and is sensitive to local indentations (lower values indicate greater indentation/non‐convexity).

#### 2.2.2 Brain Volumetrics

To investigate the relationship between brain and spinal cord morphometry, we processed all T1‐weighted images using FreeSurfer (*recon‐all*, v7.2.0) (Fischl 2012). FreeSurfer segmentations were visually quality controlled and failed or unusable outputs were excluded as previously described (Kim et al. 2026); dataset‐level terms in the normative models were used to account for scanner‐ and acquisition‐related variation in the derived volumes (Jovicich et al. 2009). For comparative analysis with spinal cord metrics, we extracted the following primary brain volumes: total brain volume, total gray matter volume, total white matter volume, thalamus volume (right + left), and brainstem volume.

To investigate cross‐sectional brain–cord relationships, we compared median lifespan trajectories and calculated Pearson correlations between observed brain and spinal cord measures within each lifespan stage. Correlations were evaluated across C1–C7 and for all cord measures; coefficients were interpreted descriptively without multiple‐comparison testing.

### 2.3 Normative Modeling and Lifespan Charting

We modeled age‐dependent normative trajectories using Generalized Additive Models for Location, Scale, and Shape (GAMLSS) (Rigby and Stasinopoulos 2005) (as implemented in R as the gamlss package), following the framework adopted for pediatric growth (Borghi et al. 2006) and recent large‐scale brain charts (Bethlehem et al. 2022; Dorfschmidt et al. 2025). Each outcome (per‐level cord morphometric or global brain volume) was fit with the Generalized Gamma (GG) family, with the location (*μ*) and scale (*σ*) parameters expressed as smooth, non‐linear functions of age and sex. Age effects were modeled with fractional polynomials (order 2 for *μ*; order 1 for *σ*), and sex entered both parameters as an additive fixed effect; no age‐by‐sex interaction was fitted. To account for between‐study/site heterogeneity, we included dataset‐level random effects in both *μ* and *σ*, yielding site‐robust population estimates while retaining within‐dataset information. In the GG family, the shape parameter (*ν*)—which governs distributional skewness—was treated as constant (i.e., not modeled as a function of covariates) in our fits. Model adequacy was assessed independently using standard GAMLSS diagnostics, including worm plots and empirical‐vs‐model centile checks, to verify that fitted curves reproduced observed trends.

Spinal cord measures were modeled in native units without intracranial‐volume normalization so that the charts describe age‐ and sex‐specific population variation and remain applicable to studies without brain imaging.

Outlier handling prior to fitting used two safeguards: (i) dataset‐ and level‐specific screening for implausible values suggestive of segmentation or coverage error, and (ii) exclusion of observations exceeding ±3 SD relative to the dataset‐level mean for that feature and level. Models were fit on the healthy cross‐sectional cohort, separately for each vertebral level (C1–C7) and morphometric feature.

The output was a set of continuous, age‐ and sex‐specific centile curves that constitute the spinal‐cord lifespan charts, describing the population distribution via *μ*, *σ* (and constant *ν*). For visualization we show canonical centiles (2nd, 5th, 10th, 25th, 50th, 75th, 90th, 95th, 98th). From the fitted median (50th‐centile) curve we derive two summaries used throughout the Results: the annualized rate of change (from the first derivative; also expressed as %/year) and the age of peak (maturation; maximum of the fitted median curve). Because these rates are derived from cross‐sectional data, they represent population‐level age associations and should not be interpreted as within‐person longitudinal change.

For interpretability, rates are summarized across predefined lifespan stages: Infancy (0–1 year), early childhood (1–6 years), late childhood (6–12 years), adolescence (12–20 years), young adulthood (20–40 years), middle adulthood (40–60 years), and late adulthood (60–100 years).

### 2.4 Model Stability and Robustness

To assess sensitivity to scan selection, we refitted the models after randomly selecting one eligible scan per participant 10 times. We also performed leave‐one‐dataset‐out refits; variability in the median centile trajectories was summarized using the coefficient of variation across refits.

### 2.5 Out‐Of‐Sample Alignment

While a new individual's morphometric measure can simply be placed on our charts (as in pediatric charting), it is advantageous to also enable a dataset‐level harmonization to account for possible scale/site/acquisition differences.

To demonstrate application of the spinal cord charts to a new study, we performed out‐of‐sample alignment of an exemplar dataset excluded from the reference‐chart used for this analysis. We chose an in‐house dataset (VUIIS‐MS) collected to study multiple sclerosis (MS), a chronic inflammatory and neurodegenerative disorder of the central nervous system in which spinal cord involvement and atrophy are common and associated with clinical disability and disease progression (Keegan et al. 2024; Bischof et al. 2022; Ruggieri et al. 2021; Lukas et al. 2021; Karthik et al. 2026; Kearney et al. 2015). T1‐weighted images were processed using the same spinal cord segmentation, vertebral labeling, quality control, and morphometric procedures used for chart construction. This cohort included 67 participants: 34 controls (age 22.7–64.1; mean age 38.6; 26 female) and 33 participants with MS (age 25.0–64.4; mean age 42.4; 29 female). We note participants with MS generally had minimal disability (mean EDSS = 2) and were on disease‐modifying treatments.

Following previous normative charting studies (Bethlehem et al. 2022; Kim et al. 2026), dataset‐specific offsets to the location and scale of the generalized gamma reference distribution were estimated from controls using maximum‐likelihood estimation. These offsets were applied unchanged to participants with MS, after which age‐ and sex‐specific centiles were calculated for every participant. The same procedure can be applied to any dataset containing age, sex, control‐group labels, and any morphometric feature.

To validate the procedure, we repeated the analysis after introducing a 5% multiplicative bias in C3 CSA. We also evaluated alignment in a larger ADNI sample of 1157 controls and 748 participants with Alzheimer disease. The reference charts, deployable model files, alignment software, and worked examples are distributed through Zenodo (https://doi.org/10.5281/zenodo.18306654).

## 3 Results

### 3.1 Non‐Linear Dynamics of Spinal Cord Morphometry Across the Lifespan

We generated normative lifespan charts for cervical spinal cord morphometry by applying GAMLSS to our large‐scale, cross‐sectional cohort. At the representative C3 level (Figure 2), the raw data and fitted centiles show nonlinear trajectories across the lifespan. CSA, AP‐diameters, and RL (transverse)‐diameters rise rapidly through childhood and adolescence, plateau in young adulthood, and decrease in later life. Other shape metrics follow different patterns: AP/RL ratio generally decreases with age, eccentricity increases modestly, and solidity is largely stable with a slight late‐life reduction.

**FIGURE 2:**
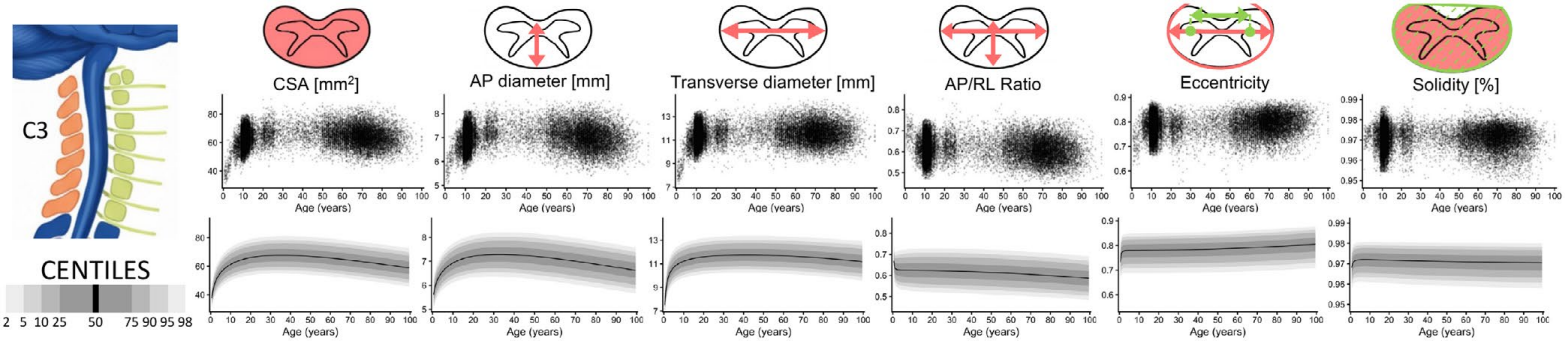
Cervical spinal cord growth charts across the lifespan. Lifespan trajectories for six morphometric features are shown at the representative C3 vertebral level. Individual data points from the healthy cohort are plotted, along with the estimated centile curves (2nd, 10th, 25th, 50th, 75th, 90th, 98th) derived from GAMLSS models. Measures of cord size, including cross‐sectional area (CSA), anterior–posterior (AP) diameter, and right–left (RL) diameter, follow a characteristic non‐linear trajectory of rapid growth through childhood, a plateau in young adulthood, and a gradual decrease in later life. In contrast, measures of cord shape exhibit distinct lifespan dynamics: AP/RL Ratio shows a subtle decline with age, Eccentricity demonstrates a gradual increase, and Solidity remains largely stable before a slight decrease in late adulthood.

Figure 3 extends these results across C1–C7 for CSA and AP diameter, presenting (i) the normative lifespan trajectory (median), (ii) the annualized rate of change derived from the first derivative, and (iii) population variability (*σ*). While the global pattern (growth → plateau → decrease) is conserved, the timing and magnitude vary by vertebral level. Parallel panels for RL diameter, AP/RL ratio, eccentricity, and solidity are provided in Figure S3, and complete centile tables for all features, levels, and sexes are provided as Supporting Information.

**FIGURE 3:**
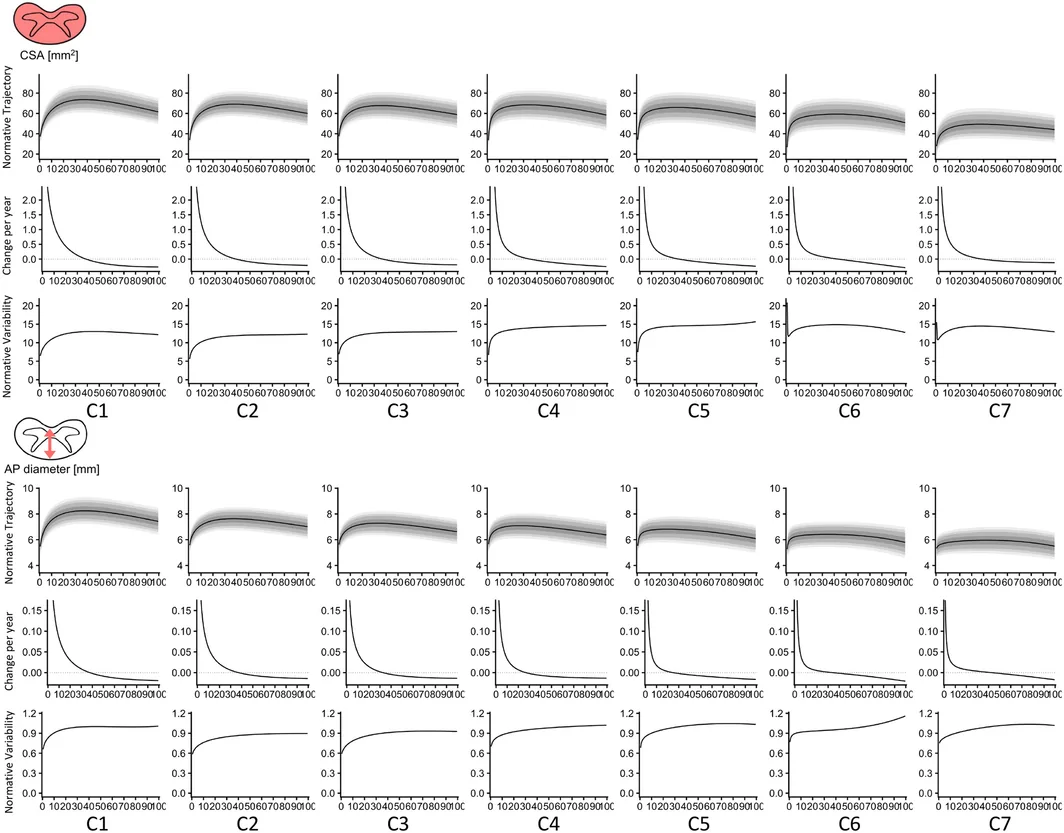
Lifespan dynamics of spinal cord morphometry across the vertebral levels figure displays the normative trajectory (centiles), annualized rate of change (change‐per‐year), and normative population variability (standard deviation, *σ*) for cross‐sectional area (CSA, top) and anterior–posterior (AP) diameter (bottom). Normative trajectories reveal distinct developmental and aging patterns that vary by vertebral level; annualized rate of change quantifies these dynamics, highlighting periods of rapid growth in childhood and adolescence followed by cross‐sectional decreases in late adulthood; Population variability illustrates the spread of measurements changes across the lifespan.

### 3.2 Regional Variations Along the Spinal Cord With Age

The normative lifespan charts reveal level‐dependent patterns across C1–C7 vertebral levels (Figure 4). Size metrics (CSA, AP, RL) generally decrease caudally, with a prominent cervical enlargement at C4–C5 driven chiefly by greater RL (transverse) diameter, consistent with neuroanatomy (Elliott 1945; Valosek, Bédard, et al. 2024). While the absolute magnitude of size metrics varies across levels, the overall shape of the lifespan trajectory—early growth and late‐life decrease—is largely conserved along the cord. In contrast, measures of cord shape, such as AP/RL ratio and eccentricity, exhibit more complex, and level‐specific lifespan dynamics, with unique patterns of development and aging at different vertebral locations.

**FIGURE 4:**
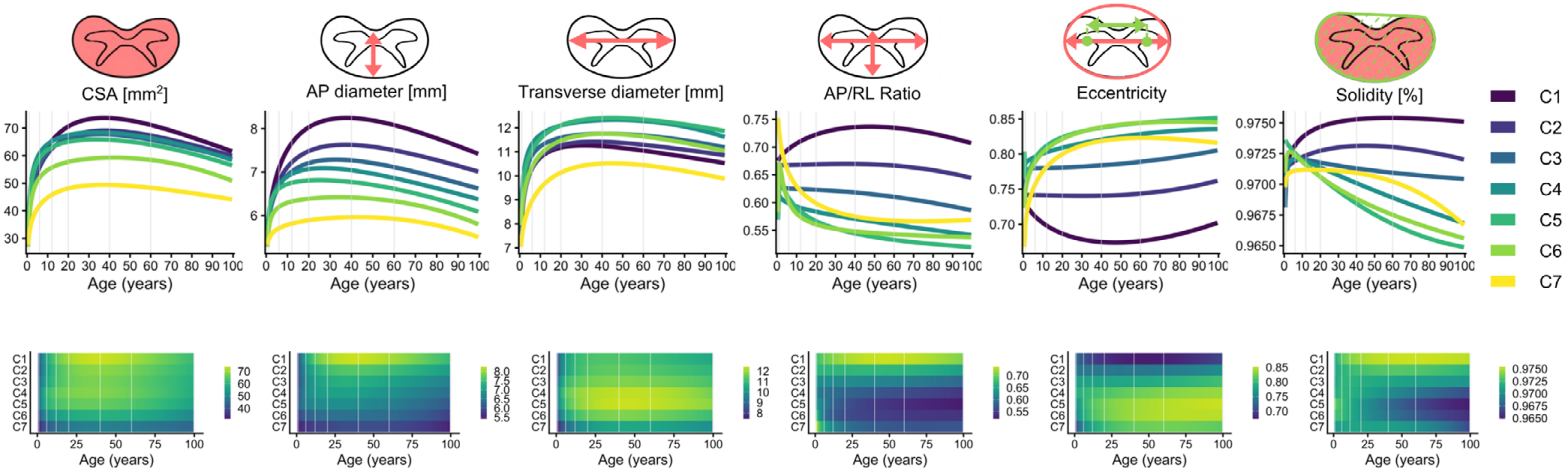
Regional variations along the spinal cord with age. Median lifespan trajectories for all six morphometric features are plotted for each cervical vertebral level (C1–C7) (top row), alongside two‐dimensional heatmaps visualizing the magnitude of each feature across both age and vertebral level (bottom row). While overall trends are shared across levels, the timing and magnitude of change are level‐dependent, and shape metrics (AP/RL ratio, eccentricity, solidity) display more heterogeneous, region‐specific dynamics.

### 3.3 Lifespan Milestones in Spinal Cord Growth and Aging

The normative lifespan charts allow for the identification of key developmental milestones, such as the age at which spinal cord morphometric measures reach their peak (Figure 5), which can be interpreted as the age of maturation (Papinutto, Asteggiano, et al. 2020). CSA peaked between 33 and 42 years across cervical levels (35 years at C3), refining a prior estimate of approximately 45 years from a smaller single‐site adult cohort (*N* = 129) (Papinutto, Asteggiano, et al. 2020). AP diameter generally peaked earlier (25–43 years) than RL diameter (31–47 years), particularly at C3–C7. These peaks provide quantitative benchmarks for the transition from growth to age‐related decline.

**FIGURE 5:**
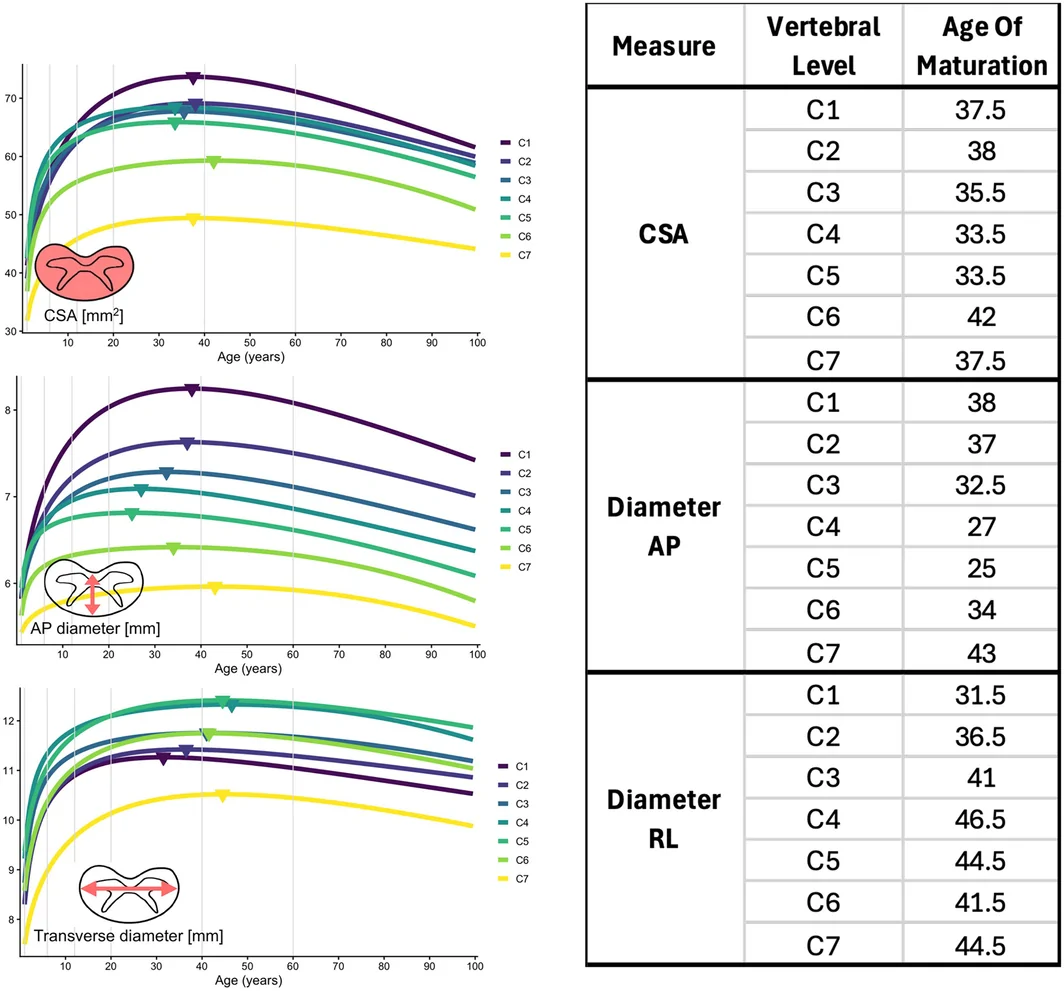
Lifespan milestones in spinal cord growth and maturation. The median lifespan trajectories for cross‐sectional area (CSA), anterior–posterior (AP) diameter, and right–left (RL) diameter are shown for each cervical vertebral level. The peak of each curve, interpreted as the age of maturation, is indicated by a marker. The peak of each curve, interpreted as the age of maturation, is marked and quantified in the accompanying table. CSA peaked between 33 and 42 years across levels, while AP diameter generally peaked earlier than RL diameter, particularly at C3–C7.

### 3.4 Quantifying Annualized Rates of Change for Clinical Benchmarking

To establish quantitative benchmarks for clinical and research applications, we derived the annualized rates of change from the (cross‐sectional) normative curves for each morphometric feature across distinct lifespan stages (Table 1). Change was greatest in early life: CSA increased dramatically in infancy and by 6.7%–9.5%/year during early childhood; over the same period, RL diameter increased more rapidly than AP diameter at every level (3.9%–5.7%/year vs. 1.1%–4.4%/year). A broad plateau followed, whereas in late adulthood CSA decreased by 0.23%–0.37%/year; AP diameter declined by 0.17%–0.22%/year, exceeding the 0.10%–0.14%/year decline in RL diameter at every cervical level. Absolute rates (mm^2^/year and mm/year) and stage‐wise results for shape metrics are provided in Table S3.

**TABLE 1:** Annualized percentage rates of cross‐sectional age‐related change in spinal cord morphometric measures.

Measure	Vertebral level	Percent change per year						
		Infancy	Early childhood	Late childhood	Adolescence	Young adulthood	Middle adulthood	Late adulthood
CSA	C1	21	7.4	2.3	0.99	0.22	−0.17	−0.37
	C2	31	8.3	2	0.83	0.19	−0.13	−0.29
	C3	26	6.9	1.6	0.66	0.12	−0.14	−0.28
	C4	54	9.1	1.2	0.43	0.065	−0.14	−0.32
	C5	50	8.4	1.1	0.4	0.058	−0.14	−0.32
	C6	75	9.5	1.2	0.47	0.14	−0.068	−0.35
	C7	29	6.7	1.5	0.63	0.14	−0.11	−0.23
Diameter AP	C1	12	4.4	1.4	0.6	0.14	−0.098	−0.22
	C2	9.5	3.4	1	0.46	0.099	−0.081	−0.17
	C3	8.3	3	0.86	0.35	0.045	−0.11	−0.18
	C4	10	2.9	0.62	0.21	−0.017	−0.13	−0.19
	C5	15	2.8	0.35	0.1	−0.026	−0.12	−0.21
	C6	14	2.5	0.34	0.13	0.022	−0.065	−0.22
	C7	3.8	1.1	0.32	0.17	0.063	−0.031	−0.19
Diameter RL	C1	20	5.1	0.97	0.33	0.031	−0.084	−0.12
	C2	21	5.2	1	0.38	0.069	−0.054	−0.099
	C3	32	5.7	0.76	0.28	0.072	−0.031	−0.11
	C4	30	5.4	0.74	0.29	0.093	−0.014	−0.14
	C5	14	3.9	0.97	0.43	0.13	−0.022	−0.1
	C6	16	4.5	1.1	0.47	0.12	−0.045	−0.13
	C7	13	4.1	1.3	0.6	0.19	−0.03	−0.14
Compression ratio	C1	0.88	0.56	0.33	0.21	0.093	−0.0075	−0.097
	C2	−0.26	−0.033	0.013	0.015	0.0057	−0.023	−0.085
	C3	−9.6	−0.86	−0.027	−0.025	−0.04	−0.064	−0.1
	C4	−1.7	−0.59	−0.22	−0.15	−0.11	−0.094	−0.085
	C5	18	0.48	−0.68	−0.39	−0.2	−0.11	−0.062
	C6	30	−0.81	−0.79	−0.31	−0.12	−0.048	−0.024
	C7	−6.1	−2.5	−0.87	−0.46	−0.21	−0.068	0.0072
Eccentricity	C1	−0.87	−0.55	−0.32	−0.21	−0.089	0.009	0.096
	C2	−0.0022	−0.0055	−0.0086	−0.0083	−0.00045	0.019	0.062
	C3	9.5	0.75	0.0098	0.01	0.019	0.032	0.052
	C4	1	0.29	0.14	0.1	0.071	0.048	0.021
	C5	−9.1	0.19	0.39	0.19	0.083	0.04	0.022
	C6	6.7	1.4	0.41	0.22	0.1	0.037	0.0023
	C7	9.8	2	0.56	0.29	0.12	0.027	−0.022
Solidity	C1	0.12	0.047	0.016	0.0081	0.0032	0.0006	−0.00078
	C2	0.019	0.013	0.0078	0.005	0.002	−0.00043	−0.0026
	C3	0.42	0.06	−0.00085	−0.0028	−0.0024	−0.0018	−0.0013
	C4	0.14	0.026	−0.0033	−0.0062	−0.0066	−0.0061	−0.0054
	C5	−0.03	−0.02	−0.015	−0.014	−0.011	−0.0086	−0.0049
	C6	0.22	0.02	−0.014	−0.012	−0.0092	−0.0068	−0.005
	C7	0.13	0.021	0.0013	−0.00003	−0.0012	−0.0036	−0.0093

### 3.5 Charting Population Variability Across the Lifespan

To characterize normative population variability, we calculated the coefficient of variation (CV) by lifespan stage (Table 2). Outside infancy, CSA CVs ranged from 8%–12% at C1–C5 and were higher at C6–C7 (13%–15%; possibly due to smaller sample sizes and increased susceptibility to algorithmic variance at the edge of the imaging field‐of‐view), while AP and RL diameter CVs remained below 10% across all levels (5.3%–9.9%). CVs were largely stable across age, with modest increases at several levels in middle and late adulthood. Stage‐wise variability (standard deviation and CV) for all features and levels is reported in Table S4.

**TABLE 2:** Population variability of spinal cord size metrics across the lifespan.

Measure	Vertebral level	Coefficient of variation						
		Infancy	Early childhood	Late childhood	Adolescence	Young adulthood	Middle adulthood	Late adulthood
CSA	C1	8.6	8.6	8.6	8.7	8.7	8.9	9.4
	C2	8.4	8.4	8.4	8.4	8.5	8.8	9.6
	C3	9.1	9.2	9.2	9.2	9.3	9.6	10
	C4	9.9	9.9	9.9	10	10	11	12
	C5	11	11	11	11	11	11	12
	C6	39	13	13	13	13	13	13
	C7	28	15	15	15	15	15	15
Diameter AP	C1	6	6	6	6	6	6.1	6.4
	C2	5.2	5.3	5.3	5.4	5.6	5.8	6.2
	C3	5.3	5.4	5.6	5.8	6.1	6.4	6.8
	C4	6.2	6.2	6.3	6.4	6.7	7	7.6
	C5	6.2	6.4	6.7	6.9	7.3	7.7	8.2
	C6	7.3	7.3	7.3	7.3	7.3	7.6	8.7
	C7	7	7.2	7.5	7.7	8.1	8.5	9
Diameter RL	C1	7.7	6	5.7	5.7	5.6	5.6	5.6
	C2	5.7	5.7	5.7	5.7	5.7	5.8	6.2
	C3	6.4	6.4	6.4	6.4	6.4	6.5	6.8
	C4	6.4	6.4	6.5	6.6	6.8	7.1	7.7
	C5	7.1	7.1	7.1	7.1	7.1	7.3	7.9
	C6	8	8	8	8	8	8.1	8.6
	C7	18	9.9	9.6	9.5	9.5	9.5	9.5

### 3.6 Sex Differences in Spinal Cord Morphometry

To characterize sex‐specific differences, we modeled lifespan trajectories for males and females by including sex as a fixed effect for both location and scale (Figure 6). Males generally had larger CSA and diameters, although the magnitude varied by feature and level, whereas shape metrics showed smaller sex effects. These findings motivate sex‐specific centiles; age‐by‐sex interactions were not evaluated.

**FIGURE 6:**
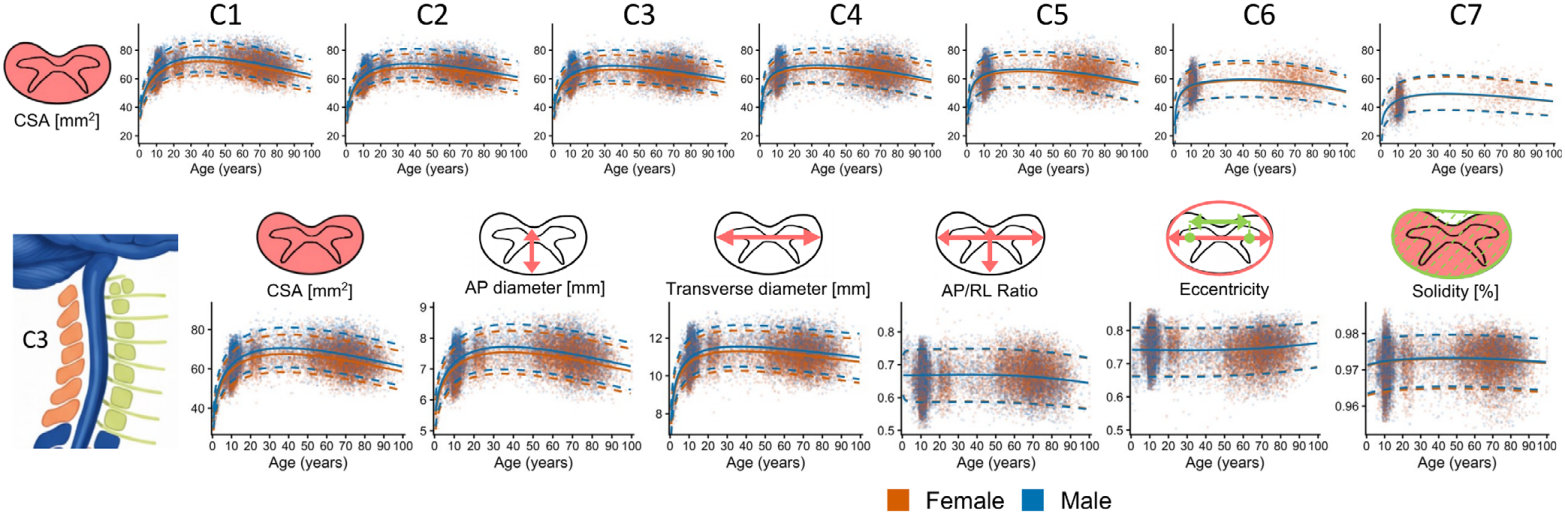
Sex‐specific lifespan trajectories of spinal cord morphometry. The top panel displays sex‐stratified centile curves (5th, 50th, 95th) for cross‐sectional area (CSA) across all seven cervical levels (C1–C7). The bottom panel shows the trajectories for all six morphometric features at the representative C3 level. In all plots, raw data points and fitted centiles are shown for males (blue) and females (red). The charts show larger cord size in males and provide sex‐specific reference distributions for individual benchmarking.

### 3.7 Brain‐Spinal Cord Relationships

To place spinal cord development and aging within the context of the entire central nervous system, we compared its lifespan trajectory with key brain volumes (Figure 7A). The timing of spinal cord growth, maturation, and aging most closely mirrors the lifespan trajectories of total white matter and brainstem volume, with gray matter, thalamic, and total brain volumes peaking earlier in the lifespan. Lifespan‐stage correlations (Figure 7B,C) are positive and strongest in childhood, decline from rostral to caudal levels, and are most robust for brainstem (followed by white matter). Shape metrics show little to no association with brain volumes. Correlation analyses excluded infancy where brain volumetry did not meet QC and age‐stage cells with insufficient sample size.

**FIGURE 7:**
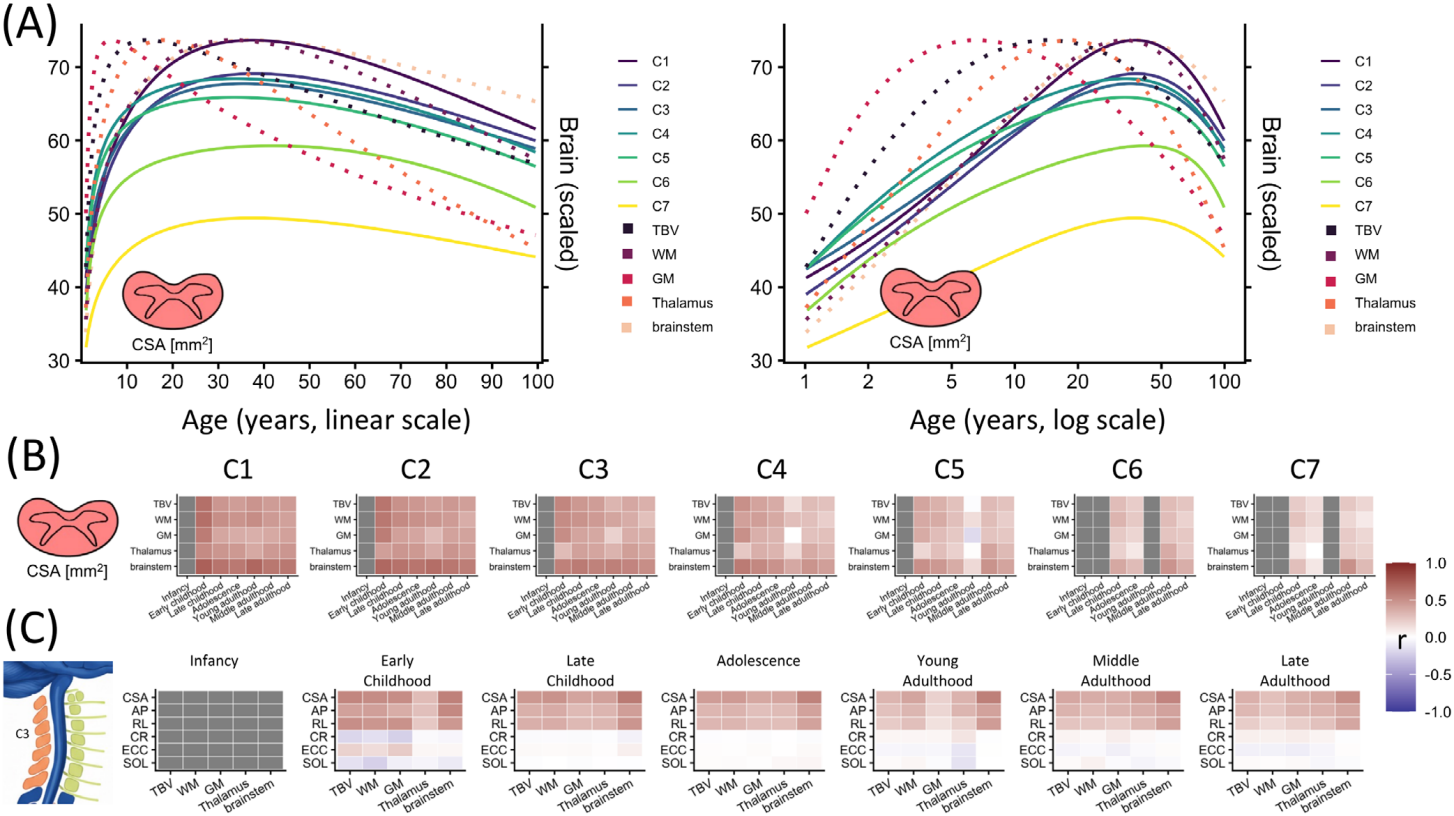
Brain‐spinal cord relationships across the lifespan. (A) The median (50th centile) lifespan trajectories for spinal cord cross‐sectional area (CSA) at all cervical levels are plotted alongside key brain morphometric volumes. Trajectories are shown on both a linear and a log‐scale age axis to accentuate developmental and aging patterns. (B) Pearson correlation coefficients between CSA and brain volumes at different lifespan stages are shown at each cervical level (C1–C7). (C) Correlations between all spinal cord features (at the representative C3 level) and brain volumes are also stratified by lifespan stage. Correlation analyses excluded infancy, as brain volumetric measurements were not consistently reliable in this age range, and any age bin with fewer than 50 subjects. Abbreviations: GM, total gray matter volume; TBV, total brain volume; WM, total white matter volume. We note x‐limit is constrained to > 0.5 years so that dynamic contrast across the lifespan is visible.

### 3.8 Aligning New Data to the Charts

Alignment of new data to the charts is shown in Figure 8. Although VUIIS‐MS showed only a modest initial offset (Figure 8A,B), alignment (Figure 8C,D) yielded mean and median control centiles of 50.4 and 48.8, respectively, with controls broadly distributed across the centile range. The same transformation provided individual centiles for participants with MS, whose mean and median centiles were lower (35.4 and 38.3), enabling subsequent analyses of clinical associations and longitudinal change relative to a normative population. A positive‐control experiment with an introduced 5% bias confirmed correction of a known dataset offset (Figure S4), while the larger ADNI example demonstrated alignment and centile extraction in a dataset with a different sample size, population, and acquisition—showing reduced SC CSA in individuals with Alzheimer's Disease, with 14% of individuals below the 5th centile (Figure S5).

**FIGURE 8:**
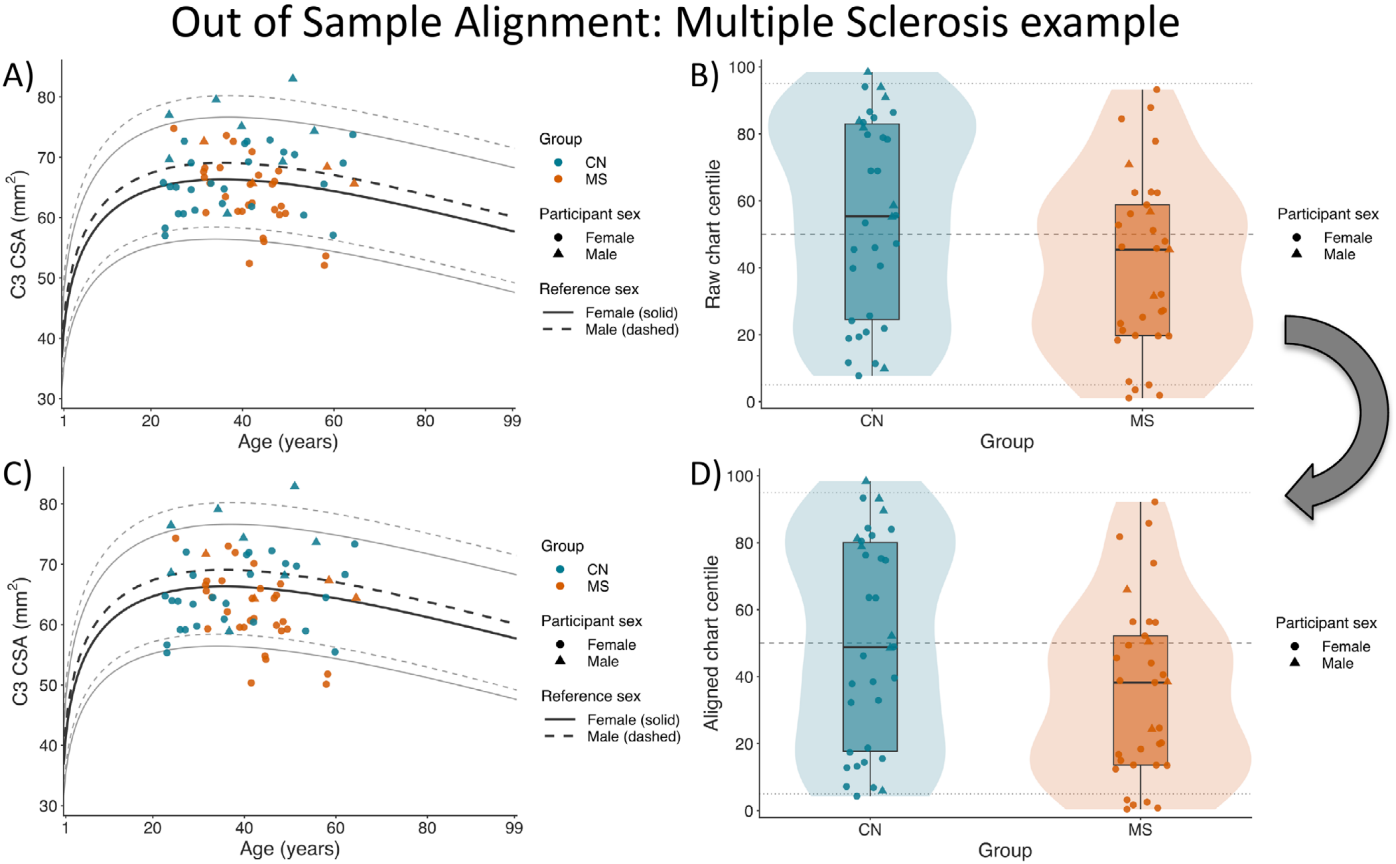
Out‐of‐sample alignment of a new dataset to the spinal cord charts. The example dataset contained 34 controls and 33 participants with multiple sclerosis (MS) and was not used to build the C3 reference chart used for this analysis. (A) Observed C3 spinal cord cross‐sectional area (CSA) plotted against the age‐ and sex‐specific reference chart. (B) Distributions of raw chart centiles in controls and participants with MS. (C) Harmonized C3 CSA after generalized gamma location and scale offsets were estimated from controls and applied unchanged to both groups. (D) Distributions of aligned chart centiles. Reference curves show the 5th, 50th, and 95th centiles; solid curves represent females and dashed curves represent males. The curved arrow indicates the transformation from raw to aligned measurements.

### 3.9 Model Stability and Robustness

Repeated scan selection and leave‐one‐dataset‐out refits produced closely overlapping median centile trajectories (Figures S6–S11). Across features, sexes, and ages at C1–C5, the median CV was 0.05% across repeated scan selections (95th percentile, 0.23%) and 0.12% across leave‐one‐dataset‐out refits (95th percentile, 0.33%); variability was greater at C6–C7 and sparsely sampled ages.

## 4 Discussion

We generated vertebral‐level, sex‐stratified normative charts for cervical spinal cord morphometry in 24,251 healthy or cognitively normal controls from 33 datasets spanning ages 0–100 years, using contrast‐agnostic segmentation (Bedard, Karthik, et al. 2025) and statistical lifespan modeling (Rigby and Stasinopoulos 2005) to harmonize measurements. These charts model the full distribution of CSA, diameters (AP, RL), and shape indices (AP/RL ratio, eccentricity, solidity) across the lifespan, revealing non‐linear trajectories—rapid early growth, a mid‐adult peak (mid‐30s for CSA), and gradual decrease—with clear level‐wise differences and stable, low relative dispersion. Together with sex‐stratified curves and brain‐cord comparisons, the resource provides a population reference that addresses long‐standing inter‐individual variability (Papinutto, Asteggiano, et al. 2020) and enables age‐ and sex‐adjusted interpretation of individual measurements.

### 4.1 Spinal Cord Charts as a Tool for Normative Assessment

Normative reference charts for the spinal cord may potentially be a powerful new tool for clinical and research applications. These charts translate absolute spinal cord measurements into age‐ and sex‐adjusted centile scores, directly paralleling pediatric growth and recent brain charts (Bethlehem et al. 2022; Habes et al. 2021). A centile expresses where an individual lies within the healthy distribution at their age/sex, clarifying whether a small CSA reflects pathology or the lower tail of typical variation. In clinical contexts, a value below a prespecified centile (e.g., 5th) may flag atypical caliber even when absolute thresholds are ambiguous, improving interpretability for disorders where cord atrophy is relevant.

Clinically, this framing helps in scenarios where variability obscures signal: (i) early disease (e.g., suspected inflammatory or neurodegenerative cord involvement) where a patient's CSA or diameter is well below age−/sex‐expected centiles despite modest absolute deviations; (ii) heterogeneous datasets (different scanners/protocols) where centiles provide a common interpretive scale; and (iii) monitoring where a drop across centiles over time suggests accelerated decline beyond age‐expected change. In each case, centiles might complement radiological assessment and absolute metrics, adding standardized context to individual measurements.

For research and trials, centile scores offer variance reduction (age/sex accounted for by design) and standardized endpoints to compare groups or track change relative to normative trajectories. Trial planners can use the stage‐specific annualized rates of change (acknowledging that these were derived from cross‐sectional data) to set realistic progression expectations and power calculations, while analyses can test whether interventions preserve centiles (or slow centile decline) versus control. In routine studies, centiles provide a common scale across sites and protocols, supporting reproducible cross‐cohort comparisons.

### 4.2 Using the Spinal Cord Charts

To facilitate practical application, we favored simplicity and accessibility; analogous to pediatric growth charts, we provide reference tables (CSVs) containing sex‐stratified centiles at 0.5‐year intervals for every morphometric feature and vertebral level. For a new individual, a feature of interest can be derived and instantly and intuitively placed at the nearest age‐ and sex‐adjusted centile. Critically, valid comparison requires that features be derived using the same processing pipeline and version to avoid methodological bias. While not a standalone diagnostic tool, this “centile scoring” enables the visualization of an individual's development or aging relative to the population, facilitating the identification of trends or extreme deviations (e.g., < 5th or > 95th percentiles).

For dataset‐level applications with suitable controls, however, we recommend control‐referenced out‐of‐sample alignment, which accounts for dataset‐specific location and scale differences before centile assignment, as in prior brain‐charting studies (Bethlehem et al. 2022; Kim et al. 2026). We demonstrated this approach in the held‐out VUIIS‐MS dataset, where participants with MS occupied descriptively lower C3 centiles than controls (Figure 8); in a positive‐control experiment that corrected an introduced 5% measurement bias (Figure S4); and in a larger held‐out ADNI cohort, where participants with Alzheimer disease occupied lower centiles and were more frequently below the 5th centile (Figure S5).

This resource builds upon previous normative efforts in the literature, representing a shift in scale and scope. Previous work by Frostell et al. (2016) provided essential aggregate reviews of segmental diameters (primarily from postmortem examinations), while (Papinutto et al. 2015; Papinutto, Asteggiano, et al. 2020; Papinutto, Cordano, et al. 2020) advanced the field by establishing variability and normalization strategies in targeted adult and pediatric cohorts. Most recently, Valosek, Bédard, et al. (2024) introduced a high‐resolution database of healthy controls (*N* = 203) standardized to the PAM50 template space. Our work complements these efforts by prioritizing sample size and lifespan coverage. Whereas Valošek et al. maximized spatial precision (slice‐wise analysis in template space) in a focused cohort, we leveraged vertebral‐level aggregation to maximize statistical power across the entire human lifespan (*N* = 24,251; ages 0–100). This trade‐off sacrifices some spatial granularity for the robust modeling of age‐related variance. Future work may aim to bridge these approaches, potentially mapping our large‐scale lifespan variance into the PAM50 coordinate system to combine high spatial fidelity with robust, data‐driven lifespan norms.

Crucially, while these charts define population‐level expectations, establishing the functional relevance of individual deviations remains a priority for future validation. Emerging evidence links cervical atrophy directly to clinical outcomes, including respiratory dysfunction in ALS (Barry et al. 2022), cognitive decline in Alzheimer's disease (Emmenegger et al. 2024), and irreversible motor disability in MS (Mahajan et al. 2025)—suggesting that ‘centile scoring’ may eventually serve as a predictive biomarker for functional impairment.

### 4.3 Biological Insights Into Lifespan Dynamics of the Spinal Cord

Our lifespan charts provide insights into the biological processes underlying spinal cord development, maturation, and aging. By quantitatively mapping the development trajectory, they revealed rapid growth in early life followed by a protracted maturation extending well into early adulthood (Lee et al. 2015). We observe that CSA and cord diameters continue to increase through adolescence, reaching peak values around the mid‐30s for CSA. This timeline suggests that the structural maturation of the human spinal cord persists considerably longer than puberty, likely driven by ongoing myelination and axonal growth during young adulthood. Indeed, extensive literature demonstrates that myelination within the central nervous system, in all association, projection, and commissural pathways, continues into the third and even fourth decades of life (Grydeland et al. 2019; Lebel et al. 2008; Lebel and Deoni 2018; Schilling et al. 2023; Brody et al. 1987; Flechsig Of Leipsic, P. 1901; Gao et al. 2009). Our finding of a peak CSA in the mid‐30s refines previous estimates from smaller adult cohorts which suggested a peak slightly later, around 45 years (Papinutto, Asteggiano, et al. 2020), potentially reflecting the enhanced temporal resolution afforded by our large‐scale dataset.

Following this peak, our charts quantify gradual reductions in cord size with aging; AP diameter declined more rapidly than RL diameter at every cervical level in late adulthood, reversing the greater RL growth seen in early childhood. This imbalance was generally accompanied by a small decrease in AP/RL ratio and increase in eccentricity, while solidity changed little. CSA and diameters are size‐based measures commonly interpreted in relation to atrophy, whereas the shape indices describe geometry and should not themselves be interpreted as direct measures of tissue loss. The underlying neurobiology of this late‐life cord atrophy likely involves a multifactorial process analogous to brain aging, including progressive demyelination, axonal loss, and neuronal shrinkage or death in spinal gray matter. This reinforces the view of the spinal cord as an integral part of the CNS aging process and suggests its metrics can act as a complement to brain measures. In summary, our charts delineate key biological milestones—the age of peak maturation and the onset and rate of age‐related decline—providing a quantitative framework for understanding spinal cord structure across the lifespan.

### 4.4 An Integrated Central Nervous System: Brain–Cord Relationships and Sex Differences

Our findings reinforce the view of the central nervous system developing and aging as an integrated unit (Geertsen et al. 2017; Kuzmicz‐Kowalska and Kicheva 2021), highlighting both coordinated brain‐cord dynamics and sex differences. We find strong positive cross‐sectional correlations between cervical cord CSA and global brain volumes (total brain, white matter, gray matter, thalamus, brainstem) (Papinutto et al. 2015; Solstrand Dahlberg et al. 2020; Engl et al. 2013), particularly during developmental periods (Figure 7B,C). This brain–cord coupling suggests an intuitive scaling effect: individuals with larger brains tend to have proportionally larger spinal cords, likely reflecting shared genetic and developmental influences on overall nervous system size (Solstrand Dahlberg et al. 2020). This relationship motivates normalization strategies that adjust cord measures for intracranial or brain volume to reduce inter‐subject variability (Bedard and Cohen‐Adad 2022; Muhammad et al. 2024; Bedard, Valošek, et al. 2025; Papinutto et al. 2015; Papinutto, Asteggiano, et al. 2020). Such approaches may improve sensitivity in some applications, but produce a different reference and require a usable brain image. Further, the lifespan trajectories show spinal cord morphometry changes closely paralleling those of brain white matter and brainstem volume (Figure 7A). This indicates that the spinal cord does not age in isolation but rather undergoes senescence in concert with the brain, again suggesting shared underlying biological mechanisms (Sastre‐Garriga et al. 2020). In pathological states like MS, this link is often more pronounced, with concurrent brain and spinal cord atrophy reflecting widespread neurodegeneration (Ruggieri et al. 2021; Khaing et al. 2023; Molazadeh et al. 2023). Understanding this normative brain‐cord relationship opens possibilities for developing combined brain‐plus‐cord centile scores as more holistic biomarkers of CNS health, aging, or disease burden, potentially identifying individuals where one component deviates unexpectedly relative to the other.

We also confirmed sex differences in spinal cord morphometry, necessitating the use of sex‐stratified normative charts (Figure 6). Males exhibited consistently larger cervical CSA and diameters than females across the lifespan. This sexual dimorphism likely reflects known differences in overall body size, spinal canal dimensions, and potentially hormonal influences on neural development. Practically, this means that comparing an individual's measurement to sex‐specific norms is necessary for accurate assessment and avoids misclassification due to inherent size differences. Our sex‐stratified charts provide this calibration, ensuring appropriate benchmarking for both males and females throughout life. The present models generate sex‐specific centile curves through fixed sex effects on distribution location and scale; future work should test whether age‐dependent changes in location, variability, or skewness differ by sex.

### 4.5 Limitations and Future Directions

While these first lifespan charts represent a significant advance for spinal cord morphometry, several limitations should be acknowledged. First, our models are derived from cross‐sectional data, which approximates lifespan trajectories but cannot capture individual longitudinal changes or potential cohort effects (Di Biase et al. 2023). Although our approach mirrors recent large‐scale brain charting initiatives (Bethlehem et al. 2022), future integration of longitudinal data is needed to fully validate these normative curves. However, our stability analyses provide confidence in the current models: sensitivity analyses support the stability of the modeling framework, demonstrating low uncertainty across alternative scan selections and robustness to the exclusion of individual datasets (Figures S6–S11).

Further limitations include the uneven sampling across age groups and lower sample sizes at caudal levels C6 and C7 (Figure 1), which reduces model precision at the extremes of the age range and lower cervical spine. For example, C7 had few (*N* = 33) individuals between 20 and 40 years of age. Moreover, because the cervical cord lies at the periphery of the field of view in standard brain MRI protocols, measurements at these caudal levels are susceptible to gradient non‐linearity distortions, a known source of morphometric variability that is particularly pronounced at the edges of the scanner field. This technical confound likely contributes to the larger coefficient of variation observed at C7 compared to rostral levels, rendering maturation and peak estimates less precise in this region. Infancy was sparsely sampled and showed elevated variability for some caudal measures; therefore, both infancy rates of change and variability estimates should be interpreted cautiously. Fitting with a log‐age range was tested, with similar results. Future work should include a more dense sampling of early and late infancy. We advocate caution and consideration when using our charts for C6/C7 levels, and for early infancy (< 1 year of age).

Methodologically, our charts are anchored to *vertebral* levels, a practical MRI landmark, rather than functional *neuronal* segments. This is particularly relevant for lifespan modeling because the spine and spinal cord do not grow at the same rate, causing the spatial correspondence between vertebral levels and underlying spinal segments to shift with age. Because of this, observed age‐related changes in morphometry at a fixed vertebral level could partially reflect this shifting anatomical frame of reference rather than solely intrinsic tissue changes. While vertebral referencing aligns with common clinical practice and is practically feasible with noninvasive clinical MRI, future work could map these norms onto segmental anatomy (Frostell et al. 2016; Valosek, Mathieu, et al. 2024) or use developmentally stable landmarks like the pontomedullary junction (Bedard and Cohen‐Adad 2022). Related to modeling, the fractional‐polynomial specification is robust but may underfit abrupt developmental transitions or more complex, multimodal lifespan trajectories. Similarly, holding the generalized gamma shape parameter constant across age, sex, and dataset may not capture changes in distributional skewness and may reduce accuracy at the extreme centiles.

Each feature was modeled independently to retain simple, interpretable centiles for individual measurements. Future work could model covariance across measures and vertebral levels to derive lower‐dimensional morphometric profiles. Next, our charts are restricted to cervical cord due to the nature of repurposed brain imaging datasets; future work can extend charting to the thoracic and lumbar cord using dedicated spine datasets. The normative values are also inherently tied to the state‐of‐the‐art contrast‐agnostic segmentation method employed; application to data processed with different algorithms may require calibration and/or harmonization. Next, averaging across vertebral levels versus slice‐wise (e.g., in PAM50 space, as in Valosek, Bédard, et al. 2024) may overshadow more subtle changes happening within a level or at disc levels (i.e., stenosis). Moreover, we covary for age and sex, but other factors could explain variance—ethnicity, size, neck length, and so forth, and it remains to be seen how best to integrate these phenotypes to identify typical versus atypical spinal cord. Finally, while site effects were statistically modeled, residual variance due to scanner hardware or specific protocols might persist (Weeda et al. 2019; Lukas et al. 2021).

The crucial next step is validating the clinical utility of these charts by applying centile scoring in patient populations (e.g., MS, SCI, DCM) to determine their sensitivity in detecting atypical development or pathological atrophy. Integrating these spinal cord charts with existing brain charts could also enable powerful, composite biomarkers for assessing overall CNS health and disease.

## 5 Conclusion

By using large‐scale brain imaging cohorts with a contrast‐agnostic segmentation approach, we have generated the first comprehensive normative charts detailing the non‐linear dynamics of cervical spinal cord morphometry across the human lifespan. These charts provide reference data on typical growth, maturation, aging, sex differences, and population variability, revealing parallel development with brain structures like white matter and the brainstem. Analogous to established growth charts, these spinal cord charts offer a framework for converting individual measurements into age‐ and sex‐adjusted centile scores, and a standardized tool to benchmark spinal cord health and detect atypical variations indicative of neurodevelopmental or neurodegenerative processes. This work lays the foundation for more precise, individualized assessment of spinal cord integrity in clinical practice and research, with the potential to enhance diagnostic accuracy, improve statistical power in clinical trials, and deepen our understanding of central nervous system development and aging. Future extensions incorporating longitudinal data, thoracic/lumbar regions, and clinical populations will further refine these essential neuroanatomical resources. The lifespan charts, model fits, and out‐of‐sample alignment code have been made available at (https://doi.org/10.5281/zenodo.18306654) to facilitate the use and application of these charts.

## Funding

This work was supported by the National Institutes of Health (K01‐EB032898, K01‐AG073584, 1R01EB017230‐01A1, K24‐AG046373, UL1‐TR000445, UL1‐TR002243, R01‐AG034962, R01‐AG056534, R01‐AG062826, R01‐HD114489).

## Conflicts of Interest

The authors declare no conflicts of interest.

## Supporting information


**Figure S1:** Spinal cord segmentation and vertebral labelling. The spinal cord toolbox contrast‐agnostic segmentation is robust to dataset, vendor, and acquisition conditions, with subjects from six cohorts shown (top). Every session was visualized twice (once with vertebral levels, and once with segmentation outline) for quality assurance.
**Figure S2:** Age and sex distributions across cervical levels. Histograms show QA‐passing healthy/control participants with a recorded age of 0–100 years in 10‐year bins, separated by sex. Participants without recorded age/sex or with age outside 0–100 years are not included. Sample sizes decrease from C1 to C7, with fewer subjects during young adulthood (20–40) and early infancy (< 1).
**Figure S3:** Lifespan dynamics of spinal cord morphometry across the cervical spine. Figure displays the normative trajectory (centiles), annualized rate of change (change‐per‐year), and normative population variability (standard deviation, *σ*) for transverse diameter, compression ratio, eccentricity, solidity. Normative trajectories reveal distinct developmental and aging patterns that vary by vertebral level; annualized rate of change quantifies these dynamics, highlighting periods of rapid growth in childhood and adolescence followed by a steady rate of atrophy in late adulthood; Population variability illustrates the spread of measurements changes across the lifespan.
**Figure S4:** Out‐of‐sample alignment following an introduced 5% measurement bias. The analysis and panel organization match Figure 8, but every C3 CSA measurement was multiplied by 1.05 before alignment. (A) Biased C3 CSA plotted against the age‐ and sex‐specific reference chart. (B) Distributions of raw chart centiles after introducing the bias. (C) Harmonized C3 CSA after offsets estimated from controls were applied to both groups. (D) Distributions of aligned chart centiles. Alignment recovered the imposed scale factor to numerical precision: the estimated change in the location offset was 0.04879016, matching log(1.05) = 0.04879016, and aligned centiles differed from the original unshifted analysis by at most 5.2 × 10^−6^ centile points. Reference curves and line styles follow Figure 8.
**Figure S5:** Out‐of‐sample alignment in the Alzheimer's Disease Neuroimaging Initiative. The example included 1157 controls and 748 participants with Alzheimer disease and was not used to build the C3 reference chart used for this analysis. (A) Observed C3 CSA plotted against the age‐ and sex‐specific reference chart. (B) Distributions of raw chart centiles in controls and participants with Alzheimer disease. (C) Harmonized C3 CSA after offsets estimated from controls were applied unchanged to both groups. (D) Distributions of aligned chart centiles. Following alignment, mean, and median centiles were 50.5 and 50.9 in controls and 43.0 and 39.9 in Alzheimer disease; 13.8% of participants with Alzheimer disease and 6.1% of controls were below the 5th centile. Reference curves and line styles follow Figure 8.
**Figure S6:** Stability analysis. Cross‐sectional datasets were randomly selected and a bootstrapping procedure was used to quantify uncertainty in models. Centiles are shown for CSA and AP diameter, across all cervical levels, with coefficient of variation (CV) < 1% for all features and levels with the exception of C7 (and in infants).
**Figure S7:** Leave‐one‐out site analysis to assess robustness of models to specific sites. Visually, centiles scores remain similar, with more site‐driven variation at C7. Left‐out datasets are shown as different colors.
**Figure S8:** Leave‐one‐out site analysis to assess robustness of models to specific sites. Visually, centiles scores remain similar, with more site‐driven variation at more caudal levels. Left‐out datasets are shown as different colors.
**Figure S9:** Leave‐one‐out site analysis to assess robustness of models to specific sites. Percent coefficient of variation across leave‐on‐out runs, for all ages. CV is typically below 1% (with exception of C7), and often below 0.3% for most features and ages, suggesting a low sensitivity to study composition.
**Figure S10:** Leave‐one‐out site analysis to assess robustness of models to specific sites. Plots show the magnitude of influences comparing each left‐out‐site to the baseline when all datasets are included. CSA, for example, shows < 0.5 mm^3^ differences due to study composition.
**Figure S11:** Leave‐one‐out site analysis to assess robustness of models to specific sites. Plots show the magnitude of influences comparing each left‐out‐site to the baseline when all datasets are included—taking the absolute value of the magnitude of differences from baseline. Intuitively, sites with larger sample sizes (ABCD, BLSA) have greater overall effects, while sites with lower ages (Humphreys) have greater influence at that lifespan stage.

**Table S1:** Datasets, sample size, and median age.

**Table S2:** T1w image cohorts, vendors, sequences.

**Table S3:** Quantifying Annualized rates of change for clinical benchmarking. Change Per Year and Percent Change Per Year are given for all features (at all levels), for each lifespan stage. Brain measurements are also given for reference.

**Table S4:** Population variability across the lifespan. Coefficient of variation, and population standard deviation are given for all features (at all levels), for each lifespan stage. Brain measurements are also given for reference.

**File S1:** Attached CSVs with sex‐stratified percentiles for all morphometric features, at all cervical levels.

## Data Availability

The lifespan charts, model fits, and out‐of‐sample alignment code have been made available at (https://doi.org/10.5281/zenodo.18306654) to facilitate the use and application of these charts. Data used in the preparation of this article were obtained from the Alzheimer's Disease Neuroimaging Initiative (ADNI) database (http://adni.loni.usc.edu/). The ADNI was launched in 2003 as a public‐private partnership, led by Principal Investigator Michael W. Weiner, MD. The primary goal of ADNI has been to test whether serial magnetic resonance imaging (MRI), positron emission tomography (PET), other biological markers, and clinical and neuropsychological assessment can be combined to measure the progression of mild cognitive impairment (MCI) and early Alzheimer's disease (AD). Data from the Alzheimer's Disease Neuroimaging Initiative (ADNI) are available upon request from https://adni.loni.usc.edu/. Data from the AOMIC‐PIOP1 dataset are freely available for download on OpenNeuro: https://openneuro.org/datasets/ds002785/versions/2.0.0. Data from the AOMIC‐PIOP2 dataset are freely available for download on OpenNeuro: https://openneuro.org/datasets/ds002790/versions/2.0.0. Data from the AOMIC‐ID1000 dataset are freely available for download on OpenNeuro: https://openneuro.org/datasets/ds003097/versions/1.2.1. Data from the Boston Adolescent Neuroimaging of Depression and Anxiety (BANDA) dataset are available upon request from https://www.humanconnectome.org/study/connectomes‐related‐anxiety‐depression. Data from BIOCARD are available upon request after filling out a data use application: https://www.gaaindata.org/partner/BIOCARD. Data from the Baltimore Longitudinal Study of Aging (BLSA) are available upon request from https://www.blsa.nih.gov/. Data from the Centre for Attention Learning and Memory (CALM) dataset are available upon request from https://calm.mrc‐cbu.cam.ac.uk/researchers/. Data from the Cambridge Center for Ageing Neuroscience (CAMCAN) dataset are available upon request from https://camcan‐archive.mrc‐cbu.cam.ac.uk/dataaccess/. Data from the Dallas Lifespan Brain Study (DLBS) dataset are freely available for download on OpenNeuro: https://openneuro.org/datasets/ds004856/versions/1.2.0. Data from the Health & Aging Brain Study—Health Disparities (HABS‐HD) dataset are available upon request from https://apps.unthsc.edu/itr/reports. Imaging data and basic demographic information for the Healthy Brain Network (HBN) dataset are freely available to download from https://fcon_1000.projects.nitrc.org/indi/cmi_healthy_brain_network/. Phenotypic data are available upon request by filling out a data use agreement: https://fcon_1000.projects.nitrc.org/indi/cmi_healthy_brain_network/Phenotypic.html. Data from the Human Connectome Project—Aging (HCPA) dataset are available upon request from https://www.humanconnectome.org/study/hcp‐lifespan‐aging. Data from the International Consortium for Brain Mapping (ICBM) dataset are available upon request from www.loni.usc.edu/ICBM. Data from the Memory and Aging Project (MAP), Religious Orders Study (ROS), and the Minority Aging Research Study (MARS) datasets are available upon request from https://www.radc.rush.edu/. Data from the Multisite, Multiscanner, and Multisubject Acquisitions for Studying Variability in Diffusion Weighted Magnetic Resonance Imaging (MASiVar) dataset are freely available for download on OpenNeuro: https://openneuro.org/datasets/ds003416/versions/2.0.2. Data from the National Alzheimer's Coordinating Center (NACC) and the Standardized Centralized Alzheimer's & Related Dementias Neuroimaging (SCAN) are available upon request from https://naccdata.org/requesting‐data/data‐request‐process. Data from the Pediatric Imaging, Neurocognition, and Genetics dataset (PING) are available for download from the National Institutes of Mental Health data archive upon request from https://nda.nih.gov/. Data from the UCLA Consortium for Neuropsychiatric Phenomics LA5c Study (UCLA) dataset are freely available for download on OpenNeuro: https://openneuro.org/datasets/ds000030/versions/1.0.0. Data from the Vanderbilt Memory and Aging Project (VMAP_JEFFERSON, VMAP_2.0) are available upon request from https://vmacdata.org/vmap/data‐requests. Data from the Vanderbilt Alzheimer's Disease Research Centre's Tennessee Alzheimer's Project (TAP) are available from https://vmacdata.org/vadrc‐tap. Data from the Wisconsin Registry for Alzheimer's Prevention (WRAP) are available upon request from https://wrap.wisc.edu/data‐requests‐2/. Data from the Adolescent Brain Cognitive Development (ABCD) Study are available for download upon request from (https://abcdstudy.org/scientists/data‐sharing/). Data from the Bipolar & Schizophrenia Consortium for Parsing Intermediate Phenotypes dataset (BSNIP1) and its renewal (BSNIP2) are available for download from the National Institutes of Mental Health data archive upon request from https://nda.nih.gov/. Vanderbilt University data (MORGAN, BABIES‐ABC, CUTTING, VUMC‐ASD) subject to third party restrictions. Please contact corresponding author for data requests.
