# Supplementary material for: Charting Cervical Spinal Cord Morphometry Across the Lifespan
Source: Hum Brain Mapp. 2026 Oct 21;47(15):e70658. doi: 10.1002/hbm.70658 (PMC13687786; doi:10.1002/hbm.70658)
Supplement: Supplementary file 1 [file HBM-47-e70658-s004.docx]

Supplementary Data and Figures for:

Charting Cervical Spinal Cord Morphometry Across the Lifespan

**Supplementary Table 1.** Datasets, sample size, and median age.

**Supplementary Figure 1.** Spinal cord segmentation and vertebral labelling. The spinal cord toolbox contrast-agnostic segmentation is robust to dataset, vendor, and acquisition conditions, with subjects from six cohorts shown (top). Every session was visualized twice (once with vertebral levels, and once with segmentation outline) for quality assurance.

**Supplementary Figure 2**. Age and sex distributions across cervical levels. Histograms show QA-passing healthy/control participants with a recorded age of 0-100 years in 10-year bins, separated by sex. Participants without recorded age/sex or with age outside 0-100 years are not included. Sample sizes decrease from C1 to C7, with fewer subjects during young adulthood (20-40) and early infancy (<1).

**Supplementary Table 2**. T1w image cohorts, vendors, sequences.

**Supplementary Data File 1**. Attached CSVs with sex-stratified percentiles for all morphometric features, at all cervical levels.

**Supplementary Figure 3**. Lifespan Dynamics of Spinal Cord Morphometry Across the Cervical Spine. Figure displays the normative trajectory (centiles), annualized rate of change (change-per-year), and normative population variability (standard deviation, σ) for Transverse Diameter, Compression Ratio, Eccentricity, Solidity. Normative trajectories reveal distinct developmental and aging patterns that vary by vertebral level; annualized rate of change quantifies these dynamics, highlighting periods of rapid growth in childhood and adolescence followed by a steady rate of atrophy in late adulthood; Population variability illustrates the spread of measurements changes across the lifespan.

**Supplementary Table 3**. Quantifying Annualized rates of change for clinical benchmarking. Change Per Year and Percent Change Per Year are given for all features (at all levels), for each lifespan stage. Brain measurements are also given for reference.

Supplementary Table 4. Population variability across the lifespan. Coefficient of variation, and population standard deviation are given for all features (at all levels), for each lifespan stage. Brain measurements are also given for reference.

**Supplementary Figure 4**. Out-of-sample alignment following an introduced 5% measurement bias. The analysis and panel organization match Figure 8, but every C3 CSA measurement was multiplied by 1.05 before alignment. (A) Biased C3 CSA plotted against the age- and sex-specific reference chart. (B) Distributions of raw chart centiles after introducing the bias. (C) Harmonized C3 CSA after offsets estimated from controls were applied to both groups. (D) Distributions of aligned chart centiles. Alignment recovered the imposed scale factor to numerical precision: the estimated change in the location offset was 0.04879016, matching log(1.05)=0.04879016, and aligned centiles differed from the original unshifted analysis by at most 5.2x10^-6 centile points. Reference curves and line styles follow Figure 8.

**Supplementary Figure 5.** Out-of-sample alignment in the Alzheimer’s Disease Neuroimaging Initiative. The example included 1,157 controls and 748 participants with Alzheimer disease and was not used to build the C3 reference chart used for this analysis. (A) Observed C3 CSA plotted against the age- and sex-specific reference chart. (B) Distributions of raw chart centiles in controls and participants with Alzheimer disease. (C) Harmonized C3 CSA after offsets estimated from controls were applied unchanged to both groups. (D) Distributions of aligned chart centiles. Following alignment, mean and median centiles were 50.5 and 50.9 in controls and 43.0 and 39.9 in Alzheimer disease; 13.8% of participants with Alzheimer disease and 6.1% of controls were below the 5th centile. Reference curves and line styles follow Figure 8.

**Supplementary Figure 6.** Stability analysis. Cross-sectional datasets were randomly selected and a bootstrapping procedure was used to quantify uncertainty in models. Centiles are shown for CSA and AP diameter, across all cervical levels, with coefficient of variation (CV) <1% for all features and levels with the exception of C7 (and in infants).

**Supplementary Figure 7.** Leave-one-out site analysis to assess robustness of models to specific sites. Visually, centiles scores remain similar, with more site-driven variation at C7. Left-out datasets are shown as different colors.

**Supplementary Figure 8**. Leave-one-out site analysis to assess robustness of models to specific sites. Visually, centiles scores remain similar, with more site-driven variation at more caudal levels. Left-out datasets are shown as different colors.

**Supplementary Figure 9.** Leave-one-out site analysis to assess robustness of models to specific sites. Percent coefficient of variation across leave-on-out runs, for all ages. CV is typically below 1% (with exception of C7), and often below 0.3% for most features and ages, suggesting a low sensitivity to study composition.

**Supplementary Figure 10.** Leave-one-out site analysis to assess robustness of models to specific sites. Plots show the magnitude of influences comparing each left-out-site to the baseline when all datasets are included. CSA, for example, shows <0.5mm^3 differences due to study composition.

**Supplementary Figure 11.** Leave-one-out site analysis to assess robustness of models to specific sites. Plots show the magnitude of influences comparing each left-out-site to the baseline when all datasets are included – taking the absolute value of the magnitude of differences from baseline. Intuitively, sites with larger sample sizes (ABCD, BLSA) have greater overall effects, while sites with lower ages (Humphreys) have greater influence at that lifespan stage.
